# Comparison of left ventricular strains and torsion derived from feature tracking and DENSE CMR

**DOI:** 10.1186/s12968-018-0485-4

**Published:** 2018-09-13

**Authors:** Gregory J. Wehner, Linyuan Jing, Christopher M. Haggerty, Jonathan D. Suever, Jing Chen, Sean M. Hamlet, Jared A. Feindt, W. Dimitri Mojsejenko, Mark A. Fogel, Brandon K. Fornwalt

**Affiliations:** 1 Department of Biomedical Engineering, University of Kentucky, Lexington, KY USA; 2 Department of Imaging Science and Innovation, Geisinger, 100 North Academy Avenue, Danville, PA 17822-4400 USA; 3 Department of Pediatrics, University of Kentucky, Lexington, KY USA; 4 Department of Electrical Engineering, University of Kentucky, Lexington, KY USA; 5 Division of Cardiology, Department of Pediatrics, Children’s Hospital of Philadelphia, Philadelphia, PA USA; 6 Department of Radiology, Geisinger, Danville, PA USA

**Keywords:** DENSE, Feature tracking, Strain, Torsion, Dyssynchrony

## Abstract

**Background:**

Cardiovascular magnetic resonance (CMR) feature tracking is increasingly used to quantify cardiac mechanics from cine CMR imaging, although validation against reference standard techniques has been limited. Furthermore, studies have suggested that commonly-derived metrics, such as peak global strain (reported in 63% of feature tracking studies), can be quantified using contours from just two frames – end-diastole (ED) and end-systole (ES) – without requiring tracking software. We hypothesized that mechanics derived from feature tracking would not agree with those derived from a reference standard (displacement-encoding with stimulated echoes (DENSE) imaging), and that peak strain from feature tracking would agree with that derived using simple processing of only ED and ES contours.

**Methods:**

We retrospectively identified 88 participants with 186 pairs of DENSE and balanced steady state free precession (bSSFP) image slices acquired at the same locations across two institutions. Left ventricular (LV) strains, torsion, and dyssynchrony were quantified from both feature tracking (TomTec Imaging Systems, Circle Cardiovascular Imaging) and DENSE. Contour-based strains from bSSFP images were derived from ED and ES contours. Agreement was assessed with Bland-Altman analyses and coefficients of variation (CoV). All biases are reported in absolute percentage.

**Results:**

Comparison results were similar for both vendor packages (TomTec and Circle), and thus only TomTec Imaging System data are reported in the abstract for simplicity. Compared to DENSE, mid-ventricular circumferential strain (Ecc) from feature tracking had acceptable agreement (bias: − 0.4%, *p* = 0.36, CoV: 11%). However, feature tracking significantly overestimated the magnitude of Ecc at the base (bias: − 4.0% absolute, *p* < 0.001, CoV: 18%) and apex (bias: − 2.4% absolute, *p* = 0.01, CoV: 15%), underestimated torsion (bias: − 1.4 deg/cm, *p* < 0.001, CoV: 41%), and overestimated dyssynchrony (bias: 26 ms, *p* < 0.001, CoV: 76%). Longitudinal strain (Ell) had borderline-acceptable agreement (bias: − 0.2%, *p* = 0.77, CoV: 19%). Contour-based strains had excellent agreement with feature tracking (biases: − 1.3–0.2%, CoVs: 3–7%).

**Conclusion:**

Compared to DENSE as a reference standard, feature tracking was inaccurate for quantification of apical and basal LV circumferential strains, longitudinal strain, torsion, and dyssynchrony. Feature tracking was only accurate for quantification of mid LV circumferential strain. Moreover, feature tracking is unnecessary for quantification of whole-slice strains (e.g. base, apex), since simplified processing of only ED and ES contours yields very similar results to those derived from feature tracking. Current feature tracking technology therefore has limited utility for quantification of cardiac mechanics.

**Electronic supplementary material:**

The online version of this article (10.1186/s12968-018-0485-4) contains supplementary material, which is available to authorized users.

## Background

Cardiac mechanics, such as strain, torsion, and dyssynchrony, are important indicators of cardiac function and independent predictors of serious outcomes, even when accounting for traditional measures such as ejection fraction [1, 2]. Several advanced cardiovascular magnetic resonance (CMR) sequences have been developed to assess cardiac mechanics including tagging [3, 4], displacement encoding with stimulated echoes (DENSE) [5–7], strain encoding (SENC) [8], and tissue phase mapping (TPM) [9]. While these techniques can provide reference standard measurements of myocardial motion and deformation, their use is often clinically impractical. Furthermore, because they are specialized non-clinical techniques, there are few large datasets available that could be used to guide the clinical use of these techniques. As such, there has been growing interest in the use of feature tracking software to approximate the mechanics produced by reference standard techniques [10–12]. While feature tracking is simple to use and requires only standard anatomical cine sequences that are widely available, it is important to assess how well measures of cardiac mechanics such as left ventricular (LV) strain, torsion, and dyssynchrony derived from feature tracking agree with those derived from reference standard techniques.

While results from feature tracking have been compared to those from tissue tagging [10, 13–16] and TPM [17], many of these studies have been limited in scope. The largest study [10], with 191 patients with Duchenne’s Muscular Dystrophy and 42 healthy controls, surveyed only mid-ventricular short-axis images, while other studies have had limited sample sizes (*n* = 18 [16], *n* = 20 [13]). Such studies have suggested that feature tracking may have poor reproducibility and poor agreement with reference standard techniques for some measures of cardiac mechanics [12, 13] due to the following potential limitations: 1) feature tracking derives displacement fields by propagating myocardial borders from frame to frame, which relies on quantification of local changes in signal intensity and therefore is likely to fail when quantifying motion parallel to or inside the myocardium where there are no features; 2) feature tracking only captures in-plane displacement, and through-plane motion of the myocardium violates the assumptions required to track pixel data.

DENSE, an advanced CMR technique, encodes a component of tissue displacement into the phase of the CMR image [5]. The reconstructed phase image measures displacements directly at the pixel-level with higher spatial resolution (2–3 mm) than myocardial tagging, which is limited by the number of tag lines that can be reliably tracked in the image. Therefore, motion within the myocardium can be accurately captured in all directions. Indeed, data from a deforming phantom demonstrated that DENSE has equal or better performance than tagged CMR, depending on the measured cardiac mechanic [18]. Several advancements in DENSE acquisition since its introduction, such as complementary spatial modulation of magnetization (CSPAMM) artifact suppression [6] and efficient spiral readouts [7], make it an ideal, highly reproducible and validated technique for reference standard measurements of myocardial motion and deformation used by numerous previous studies [18–24]. However, none of the feature tracking validation studies have been performed with DENSE. Indeed, a recent study [25] included data from both DENSE and feature tracking, but no direct comparisons were made.

Additionally, a literature review including 62 CMR feature tracking studies found that slice-wise strains (i.e. the average strain over an entire image, such as basal, mid-ventricular, or apical short-axis slices) are the most commonly reported measures derived from feature tracking (Table 1 and Additional file 1). In total, 39 studies (63%) reported either circumferential, longitudinal, or radial slice-wise strain, and 13 studies (21%) reported only those strains. However, slice-wise strains, which are reflective of the change in length of an entire contour between just two frames, end-diastole (ED) and end-systole (ES), should not require segmental motion tracking [26]. This suggests that the most commonly reported results from feature tracking could be easily assessed without performing tracking, by simply using the ED and ES contours which are already generated during most clinical CMR scans.

**Table 1:** Reported mechanics from 62 CMR feature tracking studies

	Number of Studies
Mechanics	
Circumferential Strain – slice-wise	36
Longitudinal Strain – slice-wise	28
Radial Strain – slice-wise	21
Circumferential Strain – segmental	18
Longitudinal Strain – segmental	12
Radial Strain – segmental	12
Systolic Strain Rate	5
Diastolic Strain Rate	6
Torsion	8
Torsion Rate	5
Synchrony	6
Atrial Strain	8
Right Ventricular Strain - any	13
Right Ventricular Strain - segmental	7
Other^a^	3

^a^Feature tracking in non-CMR modality

We hypothesized that LV strains, torsion, and dyssynchrony estimated from feature tracking would not agree well with those measured by DENSE as a reference standard. We also hypothesized that slice-wise strains from measuring the change in length of entire contours between the ED and ES frames (“contour-based” strains) would agree well with strains reported by feature tracking.

## Methods

### Study population

We reviewed our database of CMR participant datasets that were acquired from 2013 to 2016 at two institutions (University of Kentucky and the Children’s Hospital of Philadelphia) for all instances where both spiral cine DENSE and balanced steady state free precession (bSSFP) were acquired at the same slice location either in basal, mid-ventricular, or apical short-axis image planes or in the four-chamber image plane. The studies were approved by the local IRBs and all participants gave informed consent. During the review, no exclusions for diagnosis or the presence of cardiovascular risk factors were applied.

### Image acquisition

All datasets from the University of Kentucky were acquired on a 3 T system (Trio, Siemens Healthineers, Erlangen, Germany) while datasets from the Children’s Hospital of Philadelphia were acquired on a 1.5 T system (Avanto, Siemens Healthineers). Spiral cine DENSE images with displacements encoded in at least the two in-plane dimensions were acquired with an established spiral sequence [7, 18, 21] using the following parameters: 6 spiral interleaves with 2 spiral interleaves acquired per temporal frame, 250 × 250 to 360 × 360 mm^2^ field of view, 128 × 128 image matrix, 1.95 × 1.95 to 2.81 × 2.81 mm^2^ pixel size, 8 mm slice thickness, 1.08 ms echo time, 15 to 17 ms repetition time, 17 to 34 ms temporal resolution. Simple or balanced encoding [19] with an encoding frequency between 0.04 and 0.10 cycles/mm [20] was used to measure in-plane displacements, while through-plane dephasing [27] and CSPAMM [6] were used for echo suppression. Cine bSSFP images were acquired at the same locations as the DENSE images using the following parameters: 1.15 × 1.15 to 1.77 × 1.77 mm^2^ pixel size, 7 to 10 mm slice thickness, 1.15 to 1.51 ms echo time, 2.70 to 3.43 ms repetition time, 8 to 15 k-space segments (true number of frames, 14–30 reconstructed frames), 20.2 to 49.7 ms temporal resolution.

### DENSE strain analysis

**Fig. 1:**
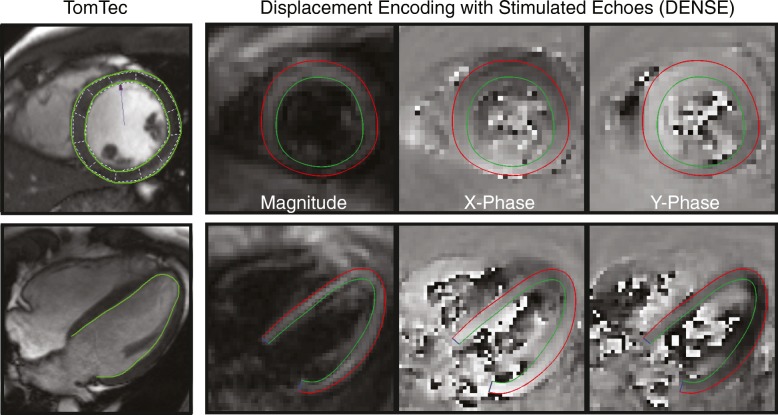
Representative images with contour overlay from feature tracking (TomTec Imaging Systems) and DENSE in mid-ventricular short-axis (top) and four-chamber (bottom) views. End-diastolic images are shown for both DENSE and feature tracking in a representative subject. In feature tracking (TomTec), only endocardial contours were used for longitudinal strain calculation. Contour-based strains were derived from the same end-diastolic/end-systolic contours exported from TomTec

Cardiac strains were derived from the DENSE images as previously described using *DENSEanalysis*, an open-source application [28] written in MATLAB (The Mathworks Inc., Natick, Massachusetts, USA) [22]. Examples of image analysis for DENSE as well as feature tracking are shown in Fig. 1. The post-processing steps for each cine DENSE slice included manual segmentation of the LV myocardium and semi-automated phase unwrapping to obtain the 2D displacements within each cardiac frame [22]. Following the unwrapping, spatial smoothing and temporal fitting of displacements (10th order polynomial) were performed as previously described to obtain smooth trajectories for all tissue points beginning at end-diastole and continuing through systole and into mid-diastole [22]. Circumferential (Ecc) and longitudinal (Ell) strains were calculated from short-axis and four-chamber images, respectively, using the Lagrangian Green finite strain tensor. Both circumferential and longitudinal strain were defined as negative for tissue shortening. For participants (*n* = 38) that had all three short-axis images (basal, mid-ventricular, and apical), cardiac torsion was calculated as the gradient of twist down the long axis of the left ventricle by finding the slope of the linear regression line between twist and longitudinal position. Twist was defined as positive for counter-clockwise rotation relative to the centroid of the LV when viewing a short-axis image from the apex towards the base. Torsion was positive when the apex was twisting more positively than the base. Dyssynchrony was quantified in these same participants (n = 38). To quantify dyssynchrony, cross-correlation delays for each segmental circumferential strain curve from the basal, mid-ventricular, and apical short-axis slices were calculated relative to a patient-specific reference curve [29]. Dyssynchrony was defined as the standard deviation of the segmental delays.

### Feature tracking strain analysis

Strain and twist were derived from bSSFP imaging with Diogenes feature tracking software (2D CPA MR, version 1.1.2.36, TomTec Imaging Systems, Munich, Germany). For short-axis images, both endocardial and epicardial contours were manually drawn at ED and the software automatically propagated the contours through the remaining frames. For the four-chamber image, only an endocardial contour was drawn before propagation, since there is minimal transmural difference in longitudinal strain (Ell) between the endocardium and epicardium as compared to the typical transmural differences seen in circumferential strain (Ecc). In the case of poor tracking, ED contours were redrawn and the propagation repeated until the tracking was visually acceptable. Ecc, Ell, and twist were derived from output files generated by the software. In short-axis slices and for appropriate comparisons to DENSE, which measures strain and twist throughout the myocardial wall, the endocardial and epicardial strains and twist from feature tracking were averaged together to obtain a single transmural value. Additional file 2 contains comparisons between just the endocardial strain from feature tracking and DENSE. Torsion and dyssynchrony were computed using the same calculations as above for DENSE imaging. Studies using feature tracking have stated that strains were derived using the 1D Lagrangian calculation [14, 30, 31], and this was reaffirmed through email correspondence with the vendor.

To assess Ecc and Ell via the change in length of entire contours, the contour position data reported in the output files for only the ED and ES frames from feature tracking using the bSSFP images were used. The frame with the smallest contour circumference was defined as the ES frame. By using these contours, rather than having an observer draw them separately, any intra- and inter-observer variability was removed for the comparison between contour-based strains and feature tracking. However, there should be no fundamental difference in manually-drawn contours and the TomTec propagated contours. This enabled a pure assessment of whether regional tracking information, which would be known to the TomTec strain calculation, is different from just using the lengths of the ED and ES contours. Contour-based strains were derived from the 1D Lagrangian strain calculation.

Finally, it is important to consider the mathematics of the strain calculations if they are different between two techniques. A full derivation of the difference between strains computed from the 2D Lagrangian Green strain tensor of DENSE and the 1D Lagrangian strain from feature tracking and contours is provided in Additional file 3. The relationship can also be found throughout the literature on deformation mechanics (e.g. see chapter 3, page 119, eq. 3.24.12 [32]). From this relationship, we propose that a correction can be applied to the 1D Lagrangian strain results to allow a proper comparison with the DENSE strain results from the 2D Lagrangian Green strain tensor. Specifically, given a 1D Lagrangian strain, ε, we propose to adjust that value by adding (1/2) ε^2^ to account for differences in the strain calculations per the following equation:

$$ 2\mathrm{D}\ \mathrm{Lagrangian}\ \mathrm{Green}\ \mathrm{Strain}=\upvarepsilon +\left(\frac{1}{2}\right){\left(\upvarepsilon \right)}^2. $$

In order to validate our findings in feature tracking, we also analyzed all data using a separate commercial feature tracking software (cvi^42^, Circle Cardiovascular Imaging Inc., Calgary, Alberta, Canada). Detailed description of data analyses and results from this comparison are shown in Additional file 4. Since the results were not substantially different between the two vendor platforms, the term “feature tracking” refers to TomTec only in the primary results below for simplification.

### Statistics

Agreement of strains and torsion between feature tracking and DENSE was assessed with Bland-Altman analyses and coefficients of variation (CoV). Based on similar analyses in previous studies [24, 33], CoVs less than 20% were interpreted as acceptable. Paired t-tests were utilized to determine whether biases were statistically significant from zero at a significance level of 0.05. Comparisons between feature tracking and DENSE were made both before and after adjusting the feature tracking results to account for the differences in strain calculations. Bland-Altman analyses and CoVs were also used to compare adjusted feature tracking strains to adjusted contour-based strains. Continuous data are presented as mean ± standard deviation.

## Results

### Study population

From the review of our database, 89 unique participants were identified that had spiral cine DENSE and bSSFP imaging at the same image locations. Of those, 1 participant had poor DENSE image quality due to aberrant prospective electrocardiogram (ECG) triggering and was therefore omitted from analyses. From these 88 participants, we obtained 186 independent image pairs, regionally distributed as follows: 39 basal short-axis, 69 mid-ventricular short-axis, 38 apical short-axis, and 40 four-chamber images. For torsion and dyssynchrony, 38 participants had all 3 of the necessary short-axis images (i.e. all participants that had an apical short-axis image also had the other short-axis images). Characteristics of the participants for each image location are reported in Table 2. Compared to other regions, there was a preponderance of healthy individuals in the four-chamber images due to only acquiring short-axis images in many patient studies.

**Table 2:** Participant characteristics

	Base (*n* = 39)	Mid (*n* = 69)	Apex/Torsion/ Dyssynchrony (*n* = 38)	Four-Chamber (*n* = 40)
Age, years	27 ± 12	26 ± 14	27 ± 12	22 ± 9
Male, n (%)	23 (59)	44 (64)	22 (58)	23 (58)
Diagnosis, n (%)				
Healthy	24 (62)	51 (74)	23 (61)	39 (98)
Tetralogy of Fallot	6 (15)	6 (9)	6 (16)	1 (3)
Duchennes	1 (3)	1 (1)	1 (3)	0 (0)
Hypertrophic CM	2 (5)	2 (3)	2 (5)	0 (0)
Ischemic CM	1 (3)	2 (3)	1 (3)	0 (0)
Other	5 (13)	7 (10)	5 (13)	0 (0)

*CM*: Cardiomyopathy

### Comparison between feature tracking and DENSE

When using just the endocardial strain from feature tracking, Ecc was significantly overestimated compared to DENSE, which measured strain throughout the myocardial wall (Additional file 2). The remainder of the Ecc, torsion, and dyssynchrony results are based on the average of the endocardial and epicardial values from feature tracking in order to better approximate the DENSE results as described in the Methods.

Before adjusting for differences in the strain calculations, Ecc was significantly overestimated by feature tracking compared to DENSE by between 2.3 and 6.0% (absolute, Table 3). Similarly, feature tracking tended to over-estimate Ell by 1.4%, although the result was not statistically significant (*p* = 0.08).

**Table 3:** Summary of strains and torsion from feature tracking and DENSE

	Feature Tracking (Unadjusted)	Feature Tracking (Adjusted)	DENSE	p_1_	p_2_
Circumferential Strain (%)					
Base	−21.7 ± 4.2	−19.3 ± 3.3	−15.2 ± 3.7	< 0.001*	< 0.001*
Mid	−19.5 ± 4.3	−17.5 ± 3.5	−17.2 ± 3.4	< 0.001*	0.36
Apex	−25.4 ± 7.8	−21.9 ± 5.7	−19.4 ± 3.6	< 0.001*	0.01*
Longitudinal Strain (%)					
Four-Chamber	−15.4 ± 5.1	−14.1 ± 4.3	−13.8 ± 2.9	0.083	0.77
Torsion (deg/cm)	2.1 ± 1.2	–	3.5 ± 0.9	< 0.001*	–
Dyssynchrony (ms)	42 ± 22	–	16 ± 20	< 0.001*	–

Unadjusted and Adjusted indicate the feature tracking results before and after adjustment, respectively

p_1_, Feature Tracking (Unadjusted) vs. DENSE; p_2_, Feature Tracking (Adjusted) vs. DENSE

*Indicates statistical significance (*p* < 0.05)

**Fig. 2:**
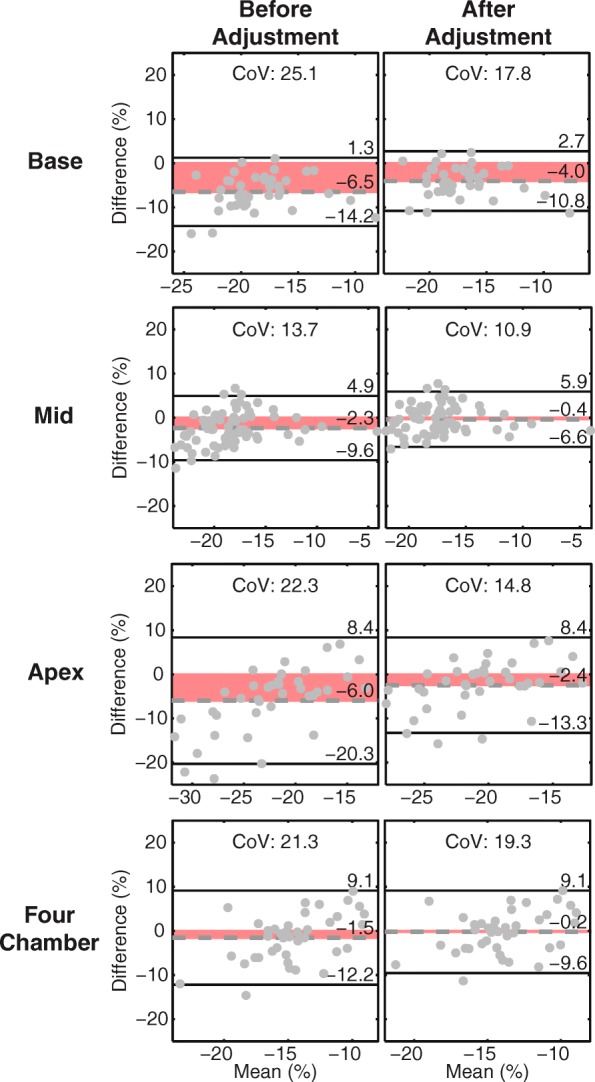
Bland-Altman analyses for circumferential and longitudinal strains between feature tracking and DENSE. Analyses were performed both before (left column) and after (right column) adjusting the feature tracking results to account for differences in the strain calculation. All differences were calculated by subtracting the DENSE strain from the feature tracking strain. All biases and 95% limits of agreement improved after adjusting the feature tracking strains. The red shaded region highlights the bias. The best agreement was observed in mid-ventricular circumferential strain. CoV, coefficient of variation

After adjusting the feature tracking results to account for differences in the strain calculations, feature tracking strains all decreased in magnitude – closer to corresponding DENSE values – such that the mid-ventricular Ecc were no longer different (−17.5 vs −17.2%, *p* = 0.36). However, basal and apical Ecc remained significantly overestimated by feature tracking even after adjustment (by 4.1 and 2.5% absolute, respectively [*p* < 0.001 for both]). A physiologic gradient of increasing Ecc magnitude from the base to the mid-ventricle to the apex was observed in the DENSE results. This gradient was not present in the feature tracking results before or after adjustment. On Bland-Altman analyses, the 95% limits of agreement and CoVs were lower after the feature tracking results were adjusted (Table 4, Fig. 2). Ecc at the mid-ventricular level had the best agreement between adjusted feature tracking and DENSE (95% limits: ±6.3%, CoV: 10.9%). All other strains demonstrated CoVs above 20% before applying the adjustment. Those same CoVs dropped below 20% after the adjustment. The CoV for Ell was 19.3%.

**Table 4:** Bland-Altman analyses and coefficients of variation comparing Feature Tracking to the reference (DENSE)

	Feature Tracking (Unadjusted) vs. DENSE			Feature Tracking (Adjusted) vs. DENSE		
	Bias	95% Limits	CoV	Bias	95% Limits	CoV
Circumferential Strain (Absolute %)						
Base	−6.5	±7.7	25.1	−4.0	±6.7	17.8
Mid	−2.3	±7.3	13.7	−0.4	±6.3	10.9
Apex	−6.0	±14.3	22.3	−2.4	±10.8	14.8
Longitudinal Strain (Absolute %)						
Four-Chamber	−1.5	±10.7	21.3	−0.2	±9.3	19.3
Torsion (deg/cm)	−1.4	±2.4	41.1	–	–	–
Dyssynchrony (ms)	26	±56	76.3	–	–	–

Unadjusted and Adjusted indicate the feature tracking results before and after adjustment, respectively

CoV indicates coefficient of variation (%)

**Fig. 3:**
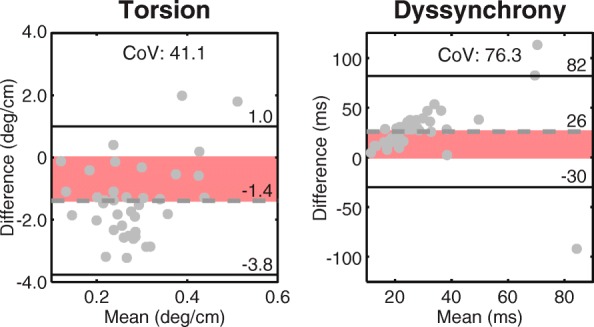
Bland-Altman analyses for torsion and dyssynchrony between feature tracking and DENSE. All differences were calculated by subtracting the DENSE measurement from the feature tracking measurement. The red shaded region highlights the bias. Poor agreement between feature tracking and DENSE was observed for both measures as demonstrated by large biases, 95% limits, and CoVs. CoV, coefficient of variation

Torsion was significantly underestimated by feature tracking compared to DENSE (2.1 vs 3.5 deg/cm, *p* < 0.001). Dyssynchrony was significantly overestimated by feature tracking (42 vs 16 ms, *p* < 0.001). Both torsion and dyssynchrony had poor agreement with DENSE as demonstrated by wide 95% limits and large CoVs (Fig. 3).

### Comparison between feature tracking and contour-based strain

**Fig. 4:**
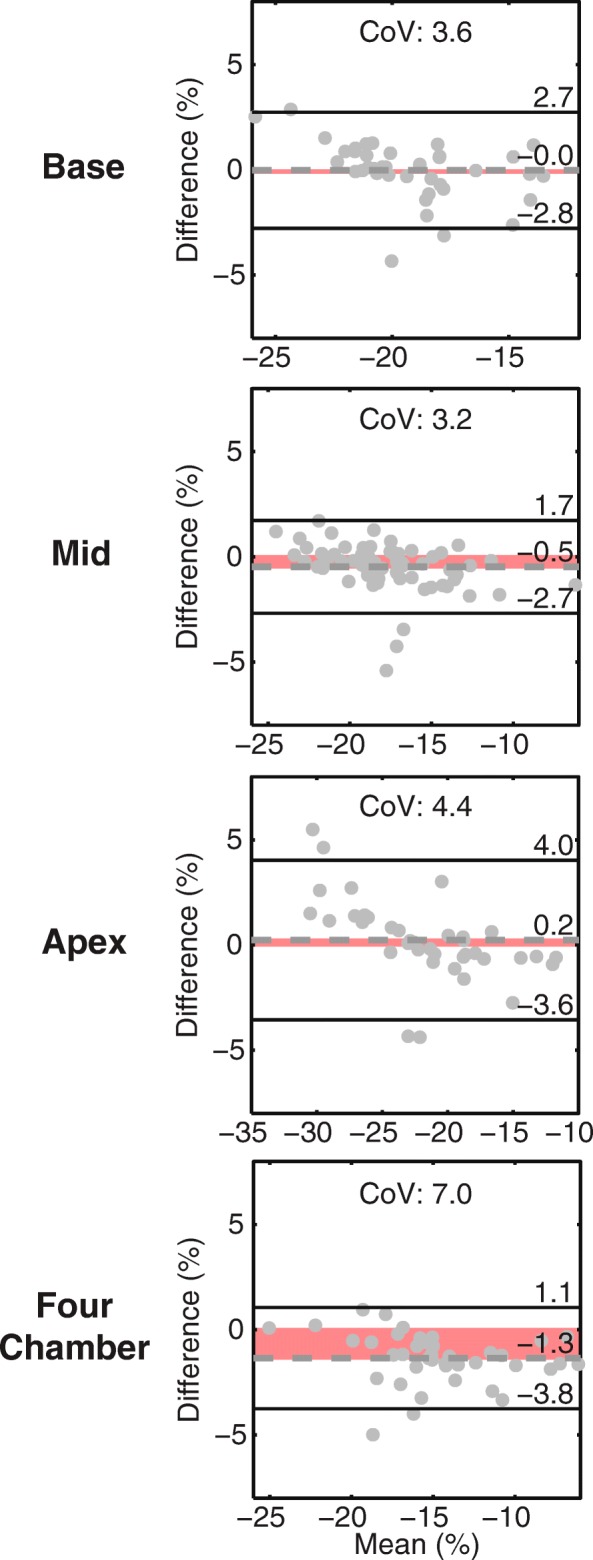
Bland-Altman analyses for circumferential and longitudinal strains between feature tracking and contour-based strains. All differences were calculated by subtracting the feature tracking strain from the contour-based strain. The red shaded region highlights the bias. Excellent agreement (small biases and tight 95% limits) was observed for all circumferential and longitudinal strains. CoV, coefficient of variation

Excellent agreement was observed between all Ecc and Ell from feature tracking and contour-based strains (Table 5, Fig. 4) with CoVs between 3.2 and 7.0%. Bland-Altman 95% limits (between ±2.2 and ± 3.8%) were substantially lower than those observed during the comparisons between feature tracking and DENSE.

**Table 5:** Bland-Altman analyses and coefficients of variation for feature tracking compared to contour-based strains

	Feature Tracking vs. Contour Strain		
	Bias	95% Limits	CoV
Circumferential Strain (Absolute %)			
Base	−0.0	±2.8	3.6
Mid	−0.5	±2.2	3.2
Apex	0.2	±3.8	4.4
Longitudinal Strain (Absolute %)			
Four-Chamber	−1.3	±2.4	7.0

CoV indicates coefficient of variation (%)

**Fig. 5:**
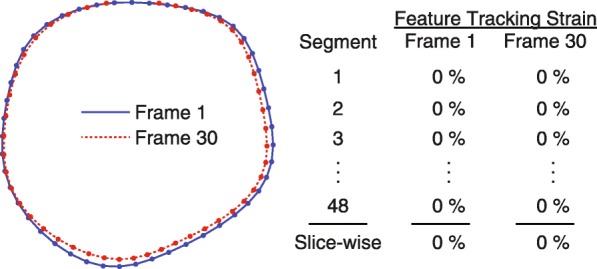
Propagated contours and reported feature tracking strains are inconsistent (representative subject). The propagated endocardial contours for frame 1 and frame 30 (the last frame) of a representative subject are shown along with the strains reported by feature tracking. Despite the differences in contour length, which would be measured as strain by the contour-based calculation, the feature tracking software reported zero strain in all segments and, thus, zero slice-wise strain. When deriving strains, the feature tracking software may employ curve-fitting *after* propagating the contours, which would lead to differences between the reported feature tracking strains and the contour-based strains

While the agreement between feature tracking and contour-based strain was excellent, we investigated why it was not perfect. Specifically, we found discrepancies between the appearance of the propagated contours and the strains that feature tracking reported for them. For example, Fig. 5 shows propagated endocardial contours for frame 1 and frame 30 (the last frame) for a short-axis image from a representative subject. There are noticeable differences in the contour lengths between those two frames, and the contour-based strain calculation would quantify a small strain for frame 30 relative to frame 1. However, the feature tracking software reported exactly zero strain for all segments in both frame 1 and frame 30, which is inconsistent with the noticeable differences between the contours.

### Validation of feature tracking using circle

In summary, the results from Circle are similar to the findings from TomTec. Consistent with TomTec, Circle significantly overestimated basal and apical Ecc, Ell, and dyssynchrony compared to DENSE (all *p* < 0.001,Additional file 4 Table S3), and the amount of overestimation was in general larger than that of TomTec (Additional file 4 Table S4, Figure S4). Only mid-ventricular Ecc from Circle showed good agreement with DENSE (bias = 0.7%, CoV = 11%). Opposite to TomTec, torsion was overestimated in Circle compared to DENSE (bias = 1.5 deg/cm, CoV = 36%). The agreements of torsion and dyssynchrony between Circle and TomTec, as well as between Circle and DENSE, were very poor with unacceptable limits and CoVs (Fig. S5). Detailed results are included in Additional file 4.

## Discussion

This study evaluated the utility of commercially available feature tracking software for quantifying measures of cardiac mechanics, including LV strains, torsion and dyssynchrony. Our primary findings included: 1) the only truly acceptable agreement between feature tracking and the reference standard (DENSE) was observed for mid-ventricular Ecc, 2) feature tracking significantly overestimated the magnitude of Ecc in basal and apical images, 3) feature tracking overestimated Ell in four-chamber images, 4) feature tracking significantly overestimated dyssynchrony and under- or over- estimated torsion depending on the vendor with unacceptable CoVs, and 5) slice-wise strains from the change in length of entire contours (contour-based strains) had excellent agreement with slice-wise strains reported by feature tracking.

### Slice-wise strains from feature tracking and contour-based strains

Feature tracking has emerged as a simple and convenient tool for estimating cardiac mechanics from standard CMR imaging. However, we found that the most commonly-reported mechanics from feature tracking (slice-wise Ecc and Ell) can be reproduced by contour-based strains. Such agreement between feature tracking and contour-based strains has been previously reported along with the suggestion that manual border delineation could be a low-cost alternative to purchasing feature tracking software [26]. Because of the excellent agreement between feature tracking and contour-based strain, regional tracking capabilities and the cost of the feature tracking software are not required to assess these metrics. Many of the insights from previous feature tracking studies could have been easily produced without the software by the manual delineation of borders at two time points, ED and ES, which is already routine for most clinical examinations in which LV volumes are reported.

However, the use of feature tracking or manual delineation to assess slice-wise strains is not beyond reproach. Only mid-ventricular Ecc had good agreement between feature tracking and DENSE with 95% limits of agreement of ±6.3% and a CoV of 10.9%. Previous studies that assessed the agreement between feature tracking and myocardial tagging have shown 95% limits ranging from ±3.3% [10] to ±9.1% [16], with several other studies in between [13, 14, 31]. However, for Ecc in basal and apical images, we found significant biases and larger CoVs, which indicates that feature tracking and DENSE do not agree as well in those regions. In particular, apical Ecc had the largest 95% limits of agreement (±10.8%), which is consistent with a previous study that also observed that the apical region had the largest 95% limits (±12.8% at 1.5 T and ±9.2% at 3 T) [16]. The largest bias (−4.0%) was observed in basal Ecc. This bias was large enough to disrupt the physiologic gradient in Ecc from base to apex that was observed in the DENSE results and has been documented extensively [21, 34–36]. These inconsistencies between feature tracking and DENSE at the basal and apical levels are likely due to both through-plane motion, which is most prominent at the base and invalidates the fundamental assumption that a segment of tissue can be observed and tracked through the entire cardiac cycle in a single 2D image plane, and the difficulty in tracking the true endocardial contour, which may be more prominent at the apex due to papillary musculature and trabeculations. Inter-test variability in both techniques, while larger in feature tracking [12, 20], also contributes to imperfect agreement between them. Among the slice-wise strains quantified in this study, Ell had the highest CoV (19.3%) along with high 95% limits of agreement compared to DENSE (±9.3%). This is consistent with a previous comparison between feature tracking and myocardial tagging which found 95% limits of agreement to be ±9.5% [13].

To reiterate, for slice-wise strains in general, manual contour delineation at just ED and ES can replace results from feature tracking. However, neither agree strongly with a reference standard technique like DENSE with the exception of mid-ventricular Ecc.

### Torsion, Dyssynchrony, and other mechanics

Less commonly-reported measures of mechanics from feature tracking include segmental strains, strain rates, torsion, and dyssynchrony. However, these are precisely the measurements for which accurate feature tracking would be most useful since none of these can be quantified by the manual delineation of contours at only ED and ES. Unfortunately, feature tracking has limited success in accurately and reproducibly quantifying these mechanics.

Previous studies of segmental strains and strain rates from feature tracking have demonstrated poor reproducibility and poor agreement with reference standards [12, 16]. Similar results were observed for torsion in the present study where torsion from feature tracking (TomTec) significantly underestimated DENSE by 1.4 deg/cm on average, while torsion from Circle overestimated DENSE by 1.5 deg./cm. This large bias is consistent with the literature as the torsion found by DENSE (3.5 ± 0.9 deg/cm) is similar to previous results from DENSE (3.1 to 3.9 deg./cm) [21] and myocardial tagging (3.4 to 3.7 deg/cm) [37] while the torsion result from TomTec (2.1 ± 1.2 deg/cm) is similar to previous feature tracking studies (2.3 ± 0.8 deg./cm) [33]. Furthermore, the CoV and 95% limits of agreement for comparing DENSE and feature tracking were high (41.1% and ± 2.4 deg/cm, respectively). Another previous study also found poor agreement and correlation between torsion derived from feature tracking and myocardial tagging as well as poor reproducibility from feature tracking [15]. The poor results from feature tracking using bSSFP images are likely due to the difficulty in tracking myocardial motion in the circumferential direction. While a strong gradient between the blood pool and the myocardium exists for accurately tracking the location of the endocardial contour in bSSFP imaging, the gradients in the orthogonal direction, which are necessary for tracking twist along that contour, are much weaker. Therefore, it is nearly impossible to track motion parallel to the myocardial wall unless additional features (such as papillary muscles) are present for tracking.

In the present study, dyssynchrony was significantly overestimated compared to DENSE (42 ± 22 vs 16 ± 20 ms) while demonstrating large 95% limits of agreement (±56 ms) and CoV (76.3%). A previous study has also demonstrated poor reproducibility for the quantification of dyssynchrony from segmental Ecc (CoV: 37.5%) [38]. The poor agreement between feature tracking and DENSE is largely due to the need for accurate segmental strains within the dyssynchrony calculation. Poor reproducibility of segmental strains, which has been demonstrated for feature tracking [12], likely resulted in both erroneously high measured dyssynchrony within volunteers (i.e. a bias) and greater variability in general.

### Limitations

While this study evaluated the agreement between measurements derived from feature tracking and those same measures derived from a reference standard DENSE sequence, we could not evaluate the prognostic utility of the measures. While we observed imperfect agreement between the techniques, it is still possible that feature tracking (or manual contour delineation) produces useful results. However, careful consideration is required before generalizing results from reference standard techniques to feature tracking. There may be cases where only a reference standard technique is sufficient (e.g., identifying a gradient in Ecc from base to apex). Measures of radial strain were not included in this study due to well-known poor reproducibility [12, 13]. In addition, the current study did not evaluate all patient populations. Different populations will likely show different levels of agreement. In particular, populations with poor function and reduced through-plane motion would be expected to have better agreement between feature tracking and reference standard techniques. However, since changes in strains may precede changes in other functional measures, quantification of cardiac strains is likely most important in populations with healthy or nearly healthy function (e.g. pediatric obesity [23]). While it is common to assess the agreement between feature tracking and reference standard techniques with only healthy participants [13], we note that there was a preponderance of healthy subjects in the assessment of Ell compared to other strains in this study.

## Conclusion

Compared to DENSE as a reference standard, feature tracking was inaccurate for quantification of apical and basal LV Ecc, Ell, torsion, and dyssynchrony. Feature tracking was only accurate for quantification of mid LV Ecc. Moreover, feature tracking is unnecessary for quantification of global peak strains, since simplified processing of only ED and ES contours yields very similar results to those derived from feature tracking. Current commercial feature tracking technology therefore has limited utility for quantification of cardiac mechanics.

## Additional files

Additional file 1: Literature Review. (DOCX 22 kb)

Additional file 2: Comparison between Endocardial Strain from Feature Tracking and DENSE. (DOCX 143 kb)

Additional file 3: Comparison of Strain Calculations. (DOCX 190 kb)

Additional file 4: Validation using a Second Feature Tracking Software. (DOCX 302 kb)

## Abbreviations

bSSFP: Balanced steady state free precession
CMR: Cardiovascular magnetic resonance
CoV: Coefficient of variation
CSPAMM: Complementary spatial modulation of magnetization
DENSE: Displacement encoding with stimulated echoes
Ecc: Circumferential strain
ED: End-diastole
Ell: Longitudinal strain
ES: End-systole
LV: Left ventricle/left ventricular
SENC: Strain encoding
TPM: Tissue phase mapping
