# Supplementary material for: Comparison of left ventricular strains and torsion derived from feature tracking and DENSE CMR
Source: J Cardiovasc Magn Reson. 2018 Sep 13;20:63. doi: 10.1186/s12968-018-0485-4 (PMC6136226; doi:10.1186/s12968-018-0485-4)
Supplement: Supplementary file 1 — Literature Review. (DOCX 22 kb) [file 12968_2018_485_MOESM1_ESM.docx]

*Literature Review*

On January 19, 2016, a PubMed search was performed to discover all studies that reported feature tracking results. 74 initial results were returned. 65 results remained after excluding reviews, editorials, and studies that did report any feature tracking results based on the consensus agreement of two reviewers (Table S1). Of these 65 results, the most common measurement was slice-wise circumferential strain (36 studies), followed by slice-wise longitudinal strain (28 studies). Other common measures included slice-wise radial strain (21 studies), segmental circumferential strain (18 studies), and any right ventricular strain (13 studies). In total, 39 studies reported either circumferential, longitudinal, or radial slice-wise strain.

Abbreviations for Table S1 include:

PMID: PubMed identification number

Ecc: Circumferential Strain

Ell: Longitudinal Strain

Err: Radial Strain

Sys. Rate: Systolic Strain Rate

Dia. Rate: Diastolic Strain Rate

Tor. Rate: Torsion Rate

Sync: Synchrony

Any RV: Any Right Ventricular Strain Measurement

Seg. RV: Segmental Right Ventricular Strain

Other: Feature Tracking in non-MRI Image Modalities

| **Table S1. Most commonly reported mechanics derived from feature tracking** | | | | | | | | | | | | | | | | | | | | | | | | | | | |  |
| --- | --- | --- | --- | --- | --- | --- | --- | --- | --- | --- | --- | --- | --- | --- | --- | --- | --- | --- | --- | --- | --- | --- | --- | --- | --- | --- | --- | --- |
|  |  | | Slice-wise  Strains | | | | Segmental Strains | | | |  | |  | |  | |  | |  | |  | |  | |  | |  |  |
| 1^st^ Author | | PMID | | *Ecc* | *Ell* | *Err* | | *Ecc* | *Ell* | *Err* | *Sys. Rate* | *Dia. Rate* | | *Torsion* | | *Tor. Rate* | | *Sync* | | *Atria* | | *Any RV* | | *Seg. RV* | | *Other* | | |
| Andre | | 25890093 | | 1 | 1 | 1 | |  |  |  |  | 1 | |  | |  | |  | |  | |  | |  | |  | | |
| Augustine | | 23331550 | | 1 | 1 | 1 | | 1 | 1 | 1 |  |  | |  | |  | |  | |  | |  | |  | |  | | |
| Baessler | | 26724669 | | 1 | 1 | 1 | |  |  |  | 1 |  | |  | |  | |  | |  | | 1 | | 1 | |  | | |
| Bhatti | | 24959555 | |  |  |  | | 1 |  |  |  |  | |  | |  | |  | |  | |  | |  | |  | | |
| Bluemke | | 26534933 | |  |  |  | |  |  |  |  |  | |  | |  | |  | |  | |  | |  | |  | | |
| Buss | | 25246506 | | 1 | 1 | 1 | |  |  |  |  |  | |  | |  | |  | |  | |  | |  | |  | | |
| Buss | | 25675901 | | 1 | 1 |  | |  |  |  |  |  | |  | |  | |  | |  | |  | |  | |  | | |
| Buss | | 24368011 | |  |  |  | |  |  |  |  |  | |  | |  | |  | |  | |  | |  | | 1 | | |
| Ceelen | | 24002783 | | 1 | 1 | 1 | | 1 | 1 | 1 | 1 | 1 | | 1 | |  | |  | | 1 | |  | |  | |  | | |
| Claus | | 26699113 | |  |  |  | |  |  |  |  |  | |  | |  | |  | |  | |  | |  | |  | | |
| Di Bella | | 20597953 | |  |  |  | |  |  |  |  |  | |  | |  | |  | |  | |  | |  | | 1 | | |
| Evin | | 25630749 | |  |  |  | |  |  |  |  |  | |  | |  | |  | | 1 | |  | |  | |  | | |
| Evin | | 26747498 | |  |  |  | |  |  |  |  |  | |  | |  | |  | | 1 | |  | |  | |  | | |
| Garg | | 25828114 | |  |  |  | |  |  |  |  |  | |  | |  | |  | |  | |  | |  | |  | | |
| Habibi | | 24813967 | |  |  |  | |  |  |  |  |  | |  | |  | |  | | 1 | |  | |  | |  | | |
| Habibi | | 26775143 | |  |  |  | |  |  |  |  |  | |  | |  | |  | | 1 | |  | |  | |  | | |
| Habibi | | 25652181 | |  |  |  | |  |  |  |  |  | |  | |  | |  | | 1 | |  | |  | |  | | |
| Habibi | | 24632272 | |  |  |  | |  |  |  |  |  | |  | |  | |  | |  | |  | |  | |  | | |
| Harrild | | 24300136 | | 1 | 1 |  | |  |  |  |  |  | |  | |  | | 1 | |  | | 1 | | 1 | |  | | |
| Hoit | | 24632275 | |  |  |  | |  |  |  |  |  | |  | |  | |  | |  | |  | |  | |  | | |
| Hor | | 21372778 | | 1 |  |  | |  |  |  |  |  | |  | |  | |  | |  | |  | |  | |  | | |
| Hor | | 20159640 | | 1 |  |  | |  |  |  |  |  | |  | |  | |  | |  | |  | |  | |  | | |
| Kawakubo | | 26384485 | |  | 1 |  | |  |  |  |  |  | |  | |  | |  | |  | | 1 | |  | |  | | |
| Kempny | | 22650308 | | 1 | 1 |  | | 1 | 1 |  |  |  | |  | |  | |  | |  | | 1 | | 1 | |  | | |
| Khan | | 25743248 | | 1 | 1 |  | | 1 | 1 |  |  |  | |  | |  | |  | |  | |  | |  | |  | | |
| Kobayashi | | 26480957 | |  |  | 1 | |  |  |  |  |  | |  | |  | |  | |  | |  | |  | |  | | |
| Kowallick | | 25196447 | |  |  |  | |  |  |  |  |  | |  | |  | |  | | 1 | |  | |  | |  | | |
| Kowallick | | 25285656 | |  |  |  | |  |  |  |  |  | | 1 | | 1 | |  | |  | |  | |  | |  | | |
| Kowallick | | 26131333 | |  |  |  | |  |  |  |  |  | |  | |  | |  | |  | |  | |  | |  | | |
| Kowallick | | 25982348 | |  |  |  | |  |  |  |  |  | |  | |  | |  | | 1 | |  | |  | |  | | |
| Kowallick | | 26114731 | |  |  |  | |  |  |  |  |  | | 1 | | 1 | |  | |  | |  | |  | |  | | |
| Kuetting | | 26426814 | |  |  |  | |  |  |  | 1 | 1 | | 1 | | 1 | |  | |  | |  | |  | |  | | |
| Kuetting | | 25467225 | |  |  |  | |  |  |  |  |  | |  | |  | | 1 | |  | |  | |  | |  | | |
| Lang | | 25976773 | | 1 |  |  | |  |  |  |  |  | |  | |  | |  | |  | |  | |  | |  | | |
| Li | | 22931981 | | 1 |  | 1 | |  |  |  |  |  | | 1 | |  | |  | |  | |  | |  | |  | | |
| Lorca | | 25476669 | |  |  |  | |  |  |  |  |  | |  | |  | |  | |  | |  | |  | |  | | |
| Lu | | 24760125 | | 1 |  | 1 | |  |  |  |  |  | |  | |  | |  | |  | |  | |  | |  | | |
| Lu | | 23497777 | | 1 | 1 |  | |  |  |  |  |  | |  | |  | |  | |  | | 1 | |  | |  | | |
| Lurz | | 25944047 | | 1 |  | 1 | | 1 |  | 1 |  |  | |  | |  | |  | |  | |  | |  | |  | | |
| Maret | | 19917130 | |  |  |  | |  | 1 | 1 |  |  | |  | |  | |  | |  | |  | |  | |  | | |
| Meyer | | 24906997 | |  | 1 | 1 | |  | 1 | 1 |  |  | |  | |  | |  | |  | |  | |  | |  | | |
| Miszalski-Jamka | | 23212274 | |  |  |  | | 1 | 1 | 1 |  |  | |  | |  | |  | |  | |  | |  | |  | | |
| Moody | | 24677420 | | 1 | 1 |  | |  |  |  | 1 | 1 | |  | |  | |  | |  | |  | |  | |  | | |
| Moon | | 25727084 | | 1 | 1 |  | |  |  |  |  |  | |  | |  | | 1 | |  | | 1 | |  | |  | | |
| Morton | | 22721175 | | 1 | 1 | 1 | | 1 | 1 | 1 |  |  | |  | |  | |  | |  | | 1 | | 1 | |  | | |
| Nucifora | | 26210792 | | 1 |  |  | | 1 |  |  | 1 | 1 | |  | |  | |  | |  | |  | |  | |  | | |
| Nucifora | | 24993044 | |  |  |  | |  |  |  |  |  | | 1 | | 1 | |  | |  | |  | |  | |  | | |
| Obokata | | 26377901 | | 1 | 1 | 1 | |  |  |  |  |  | |  | |  | |  | |  | |  | |  | |  | | |
| Onishi | | 25577185 | | 1 | 1 |  | |  |  |  |  |  | |  | |  | |  | |  | |  | |  | |  | | |
| Onishi | | 24134158 | |  |  |  | |  |  |  |  |  | |  | |  | | 1 | |  | |  | |  | |  | | |
| Orwat | | 26715570 | | 1 | 1 | 1 | |  |  |  |  |  | |  | |  | |  | |  | | 1 | |  | |  | | |
| Padiyath | | 23167248 | | 1 | 1 | 1 | |  |  |  |  |  | |  | |  | |  | |  | | 1 | |  | |  | | |
| Prati | | 26534932 | |  |  |  | |  |  |  |  |  | |  | |  | | 1 | |  | | 1 | | 1 | |  | | |
| Riffel | | 25643953 | | 1 | 1 |  | |  |  |  |  |  | |  | |  | |  | |  | |  | |  | |  | | |
| Roujol | | 24798588 | |  |  |  | |  |  |  |  |  | |  | |  | |  | |  | |  | |  | |  | | |
| Sampaio | | 26187817 | | 1 | 1 | 1 | |  |  |  |  |  | |  | |  | |  | |  | |  | |  | |  | | |
| Schmidt | | 24373377 | | 1 | 1 |  | |  |  |  |  |  | |  | |  | |  | |  | |  | |  | |  | | |
| Schneeweis | | 25803969 | | 1 |  |  | | 1 |  |  |  |  | | 1 | | 1 | |  | |  | |  | |  | |  | | |
| Schneeweis | | 25316531 | |  |  |  | | 1 |  |  |  |  | |  | |  | |  | |  | |  | |  | |  | | |
| Schuster | | 21992220 | | 1 | 1 | 1 | |  |  |  |  |  | |  | |  | |  | |  | | 1 | |  | |  | | |
| Schuster | | 23246014 | | 1 | 1 | 1 | | 1 | 1 | 1 |  |  | |  | |  | |  | |  | | 1 | | 1 | |  | | |
| Schuster | | 25848764 | |  |  |  | | 1 |  | 1 |  |  | |  | |  | |  | |  | |  | |  | |  | | |
| Schuster | | 22130224 | | 1 |  | 1 | | 1 |  | 1 |  |  | |  | |  | |  | |  | |  | |  | |  | | |
| Shetye | | 26730301 | |  |  |  | |  |  |  |  |  | |  | |  | |  | |  | |  | |  | |  | | |
| Singh | | 24700404 | | 1 | 1 |  | |  |  |  |  | 1 | |  | |  | |  | |  | |  | |  | |  | | |
| Smith | | 25159233 | | 1 | 1 | 1 | | 1 | 1 | 1 |  |  | |  | |  | |  | |  | |  | |  | |  | | |
| Smith | | 24513464 | | 1 | 1 | 1 | | 1 | 1 | 1 |  |  | |  | |  | |  | |  | |  | |  | |  | | |
| Taylor | | 25711353 | | 1 | 1 | 1 | |  |  |  |  |  | |  | |  | |  | |  | |  | |  | |  | | |
| Taylor | | 24852836 | |  |  |  | |  |  |  |  |  | |  | |  | | 1 | |  | |  | |  | |  | | |
| Tee | | 25853636 | |  |  |  | |  |  |  |  |  | |  | |  | |  | |  | |  | |  | | 1 | | |
| Vigneault | | 26497822 | |  |  |  | |  |  |  |  |  | |  | |  | |  | |  | | 1 | | 1 | |  | | |
| Weigand | | 26717912 | |  |  |  | | 1 | 1 |  |  |  | |  | |  | |  | |  | |  | |  | |  | | |
| Wu | | 24450803 | |  |  |  | | 1 |  |  |  |  | |  | |  | |  | |  | |  | |  | |  | | |
| Zhou | | 26363710 | | 1 |  |  | |  |  |  |  |  | | 1 | |  | |  | |  | |  | |  | |  | | |
| **Total** | | -- | | 36 | 28 | 21 | | 18 | 12 | 12 | 5 | 6 | | 8 | | 5 | | 6 | | 8 | | 13 | | 7 | | 3 | | |
