# Supplementary material for: Comparison of left ventricular strains and torsion derived from feature tracking and DENSE CMR
Source: J Cardiovasc Magn Reson. 2018 Sep 13;20:63. doi: 10.1186/s12968-018-0485-4 (PMC6136226; doi:10.1186/s12968-018-0485-4)
Supplement: Supplementary file 2 — Comparison between Endocardial Strain from Feature Tracking and DENSE. (DOCX 143 kb) [file 12968_2018_485_MOESM2_ESM.docx]

*Comparison between Endocardial Strain from Feature Tracking and DENSE*

Circumferential strain at all image slices was significantly overestimated by feature tracking compared to DENSE when just the endocardial strain from feature tracking was used (Table S2, Figure S1). After adjusting for differences in the strain calculations, feature tracking strains decreased in magnitude and the amount of overestimation was reduced but not eliminated. The remaining differences are largely due to DENSE measuring strain throughout the myocardial wall compared to just the endocardial contour from feature tracking.

| **Table S2. Summary of circumferential strain from feature tracking (endocardial) and DENSE** | | | | | | |  |
| --- | --- | --- | --- | --- | --- | --- | --- |
|  | Feature Tracking (Unadjusted) | | Feature Tracking (Adjusted) | DENSE | P_1_ | P_2_ | |
| Circumferential Strain (%) | |  |  |  |  |  | |
| *Base* | -29.9 ± 5.2 | | --25.3 ± 3.7 | -15.2 ± 3.7 | <0.001* | <0.001* | |
| *Mid* | -26.7 ± 5.7 | | --23.0 ± 4.3 | -17.2 ± 3.4 | <0.001* | <0.001* | |
| *Apex* | -34.8 ± 10.6 | | -28.2 ± 6.6 | -19.4 ± 3.6 | <0.001* | <0.001* | |
| Unadjusted and Adjusted indicate the feature tracking results before and after adjustment, respectively | | | | | | | |
| P_1_, Feature Tracking (Unadjusted) vs. DENSE; P_2_, Feature Tracking (Adjusted) vs. DENSE  *Indicates statistical significance (P < 0.05) | | | | | | | |


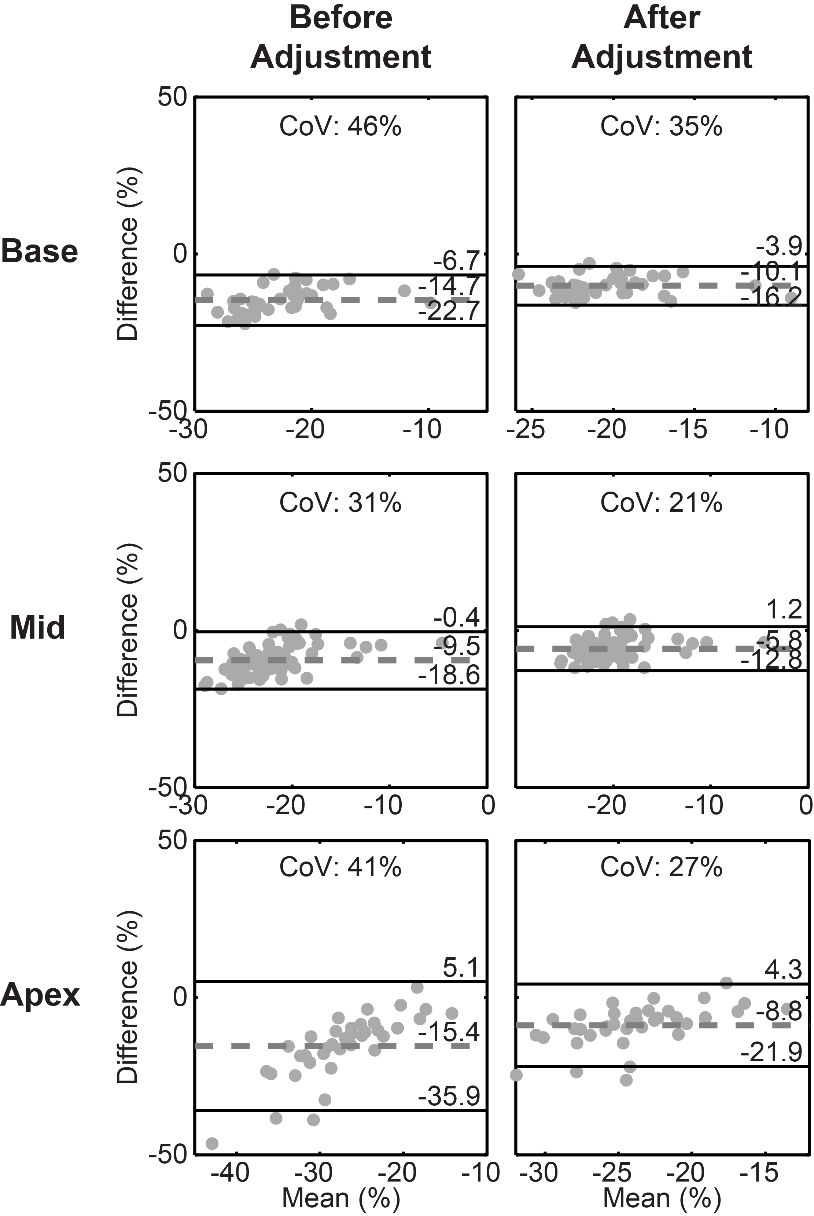


**Figure S1. Bland-Altman analyses for circumferential between feature tracking (endocardial) and DENSE.** Analyses were performed both before (left column) and after (right column) adjusting the feature tracking results. All differences were calculated by subtracting the DENSE strain from the feature tracking strain. All biases and 95% limits of agreement improved after adjusting the feature tracking strains. The large negative biases indicate that feature tracking with just the endocardial contour overestimates circumferential strain compared to DENSE for all image slices.
