# Supplementary material for: Comparison of left ventricular strains and torsion derived from feature tracking and DENSE CMR
Source: J Cardiovasc Magn Reson. 2018 Sep 13;20:63. doi: 10.1186/s12968-018-0485-4 (PMC6136226; doi:10.1186/s12968-018-0485-4)
Supplement: Supplementary file 3 — Comparison of Strain Calculations. (DOCX 190 kb) [file 12968_2018_485_MOESM3_ESM.docx]

*Comparison of Strain Calculations*

The 1D Lagrangian strain calculation (ε_L_) has been well-described as the change in the length (ΔL) of a segment of tissue divided by its initial length (L_0_):

${}_{L}=\frac{\Delta L}{L_{0}}$ ( 1 )

The differences between this common calculation and another common 1D calculation, natural strain (ε_N_), have also been well-documented as natural strain is related to Lagrangian strain through the natural logarithm (ln) [1]:

${}_{N}=ln\left( {}_{L}+1 \right)$ ( 2 )

However, in 2 or more dimensions, it is not common or appropriate to use 1-dimensional calculations. Indeed, given the large, finite deformations that occur within the heart, it is common to use the Lagrangian Green finite strain tensor. This tensor, which has been used throughout the DENSE literature and in myocardial tagging literature [2–6], relies on spatial derivatives of the displacement field. The relationship between the 1D and 2D calculations has not previously been described in cardiac strain literature [7].


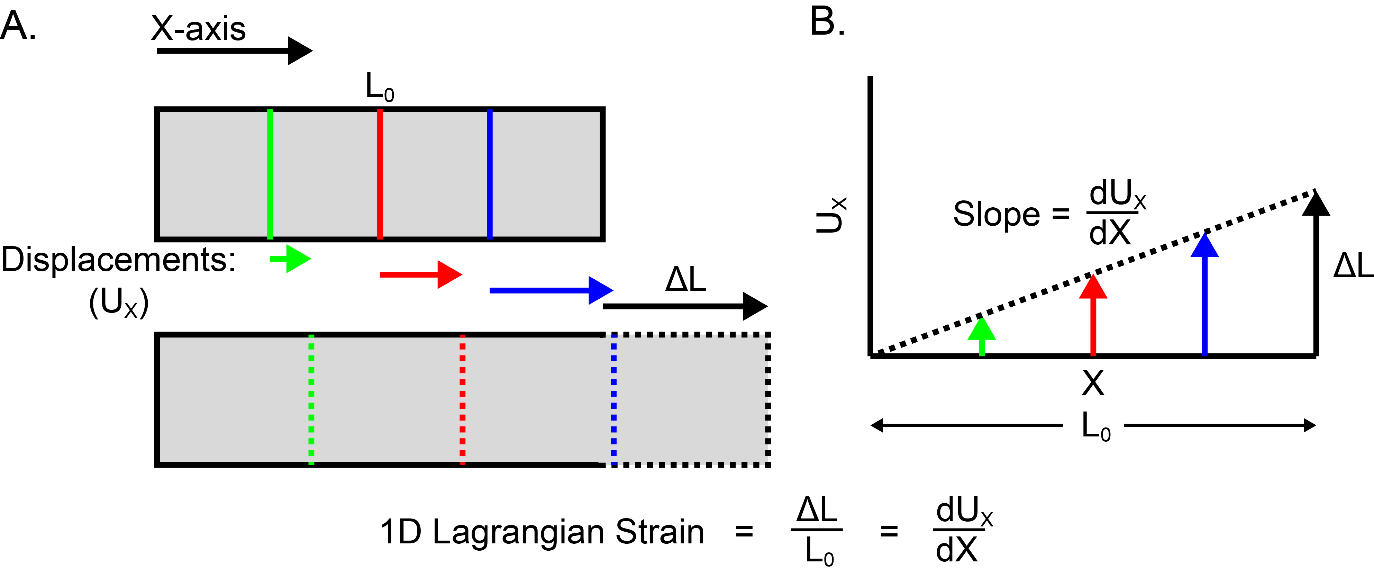


**Figure S2. 1D Lagrangian strain is the change in length over initial length or the spatial derivative of displacement. (A)** A piece of tissue with initial length L_0_ is lengthened by ΔL. 1D Lagrangian strain can be calculated as the ratio of ΔL to L_0_. Alternatively, the displacements within the piece of tissue can be considered. **(B)** The derivative (slope) of those displacements with respect to their initial locations is an equivalent calculation of 1D Lagrangian strain.

In order to compare the 1D and 2D calculations, it is necessary to consider the 1D Lagrangian strain calculation as a spatial derivative of displacement (Figure 1). As a derivative, the 1D Lagrangian strain is given by:

${}_{L}=\frac{dU_{x}}{dX}$ ( 3 )

Where U_x_ is the displacement in the x-direction. Then, the 2D calculation can be considered in two steps. First, the deformation gradient tensor (F) is formed from four spatial derivatives of the displacement field and the identity matrix (I):

$F=\left[ \begin{matrix} \frac{dU_{x}}{dX} & \frac{dU_{x}}{dY} \\ \frac{dU_{y}}{dX} & \frac{dU_{y}}{dY} \end{matrix} \right]+I$ ( 4 )

Second, the Lagrangian Green finite strain tensor (E) is calculated by the following matrix equation where superscript “T” denotes the transpose operation:

$E=\left( \frac{1}{2} \right)\left( F^{T}F-I \right)$ ( 5 )

For comparison with the 1D calculation, Lagrangian Green strain (ε_G_) in the x-direction is given by the first component of E:

${}_{G}=\frac{dUx}{dX}+\left( \frac{1}{2} \right)\left( \frac{dU_{x}}{dX} \right)^{2}+\left( \frac{1}{2} \right)\left( \frac{dU_{y}}{dX} \right)^{2}$ ( 6 )

By inspection of the terms in ε_G_, the first term is equal to the 1D Lagrangian strain. The second term is half of the square of the 1D Lagrangian strain, which would be a negligible component *if* the strain is infinitesimal. The final term is half of the square of a shear component, which is negligible if the amount of shear is infinitesimal. Ignoring the shear component, the relationship between the 2D calculation (ε_G_) and 1D calculation (ε_L_) is:

${}_{G}={}_{L}+\left( \frac{1}{2} \right)\left( {}_{L} \right)^{2}$ ( 7 )

This relationship is shown graphically in Figure 2. For negative strains, such as circumferential and longitudinal strains, the magnitude of the 2D calculation is lower than the 1D calculation. However, for positive strains, such as radial strain, the 2D calculation results in a higher magnitude strain.


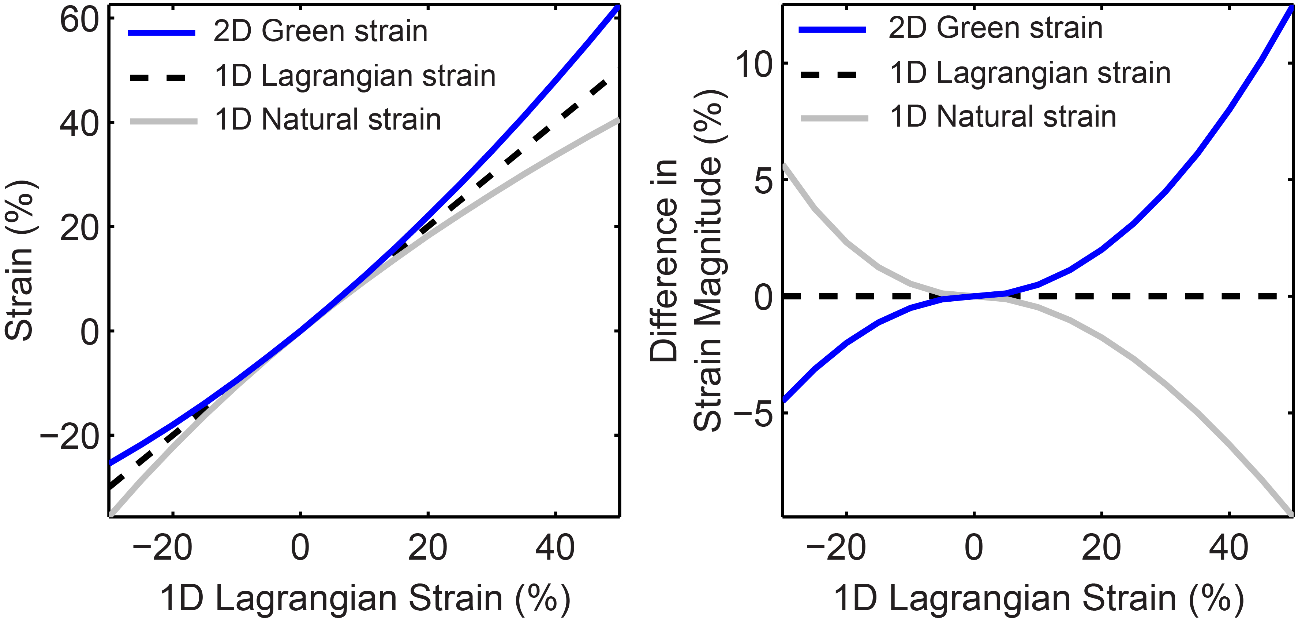


**Figure S3. Relationships between 2D and 1D strain calculations. (Left)** 2D Green strain and 1D natural strain are shown as a function of 1D Lagrangian strain. Both of them deviate from the 1D Lagrangian strain when the strain is not near zero. **(Right)** The differences in strain *magnitude* relative to the 1D Lagrangian strain magnitude are shown. For negative strains, such as circumferential or longitudinal strains, the 2D Green strain calculation results in a lower magnitude strain. The opposite is true for positive strains (such as radial strain). The relationship between 2D Green strain and 1D Lagrangian strain is opposite to that between 1D natural strain and 1D Lagrangian strain.

In order to properly evaluate the agreement between techniques that report 1D Lagrangian strain (such as feature tracking or contour-based strains) and reference standard techniques that use the 2D Lagrangian Green strain tensor (such as DENSE), we propose that a correction can be applied to the 1D strain results based on the above relationship. Specifically, given a 1D Lagrangian strain ε_L_, we propose to adjust that value by adding (1/2)( ε_L_)^2^ to account for the differences between the strain calculations.

**References**

1. Voigt J-U, Pedrizzetti G, Lysyansky P, Marwick TH, Houle H, Baumann R, Pedri S, Ito Y, Abe Y, Metz S, Song JH, Hamilton J, Sengupta PP, Kolias TJ, D’Hooge J, Aurigemma GP, Thomas JD, Badano LP: **Definitions for a Common Standard for 2D Speckle Tracking Echocardiography: Consensus Document of the EACVI/ASE/Industry Task Force to Standardize Deformation Imaging**. *J Am Soc Echocardiogr* 2015, **28**:183–193.

2. Zhong X, Spottiswoode BS, Meyer CH, Kramer CM, Epstein FH: **Imaging three-dimensional myocardial mechanics using navigator-gated volumetric spiral cine DENSE MRI.** *Magn Reson Med* 2010, **64**:1089–97.

3. Moore CC, Lugo-Olivieri CH, McVeigh ER, Zerhouni E a: **Three-dimensional systolic strain patterns in the normal human left ventricle: characterization with tagged MR imaging.** *Radiology* 2000, **214**:453–66.

4. Auger D a, Zhong X, Epstein FH, Spottiswoode BS: **Mapping right ventricular myocardial mechanics using 3D cine DENSE cardiovascular magnetic resonance.** *J Cardiovasc Magn Reson* 2012, **14**:4.

5. Spottiswoode BS, Zhong X, Hess a T, Kramer CM, Meintjes EM, Mayosi BM, Epstein FH: **Tracking myocardial motion from cine DENSE images using spatiotemporal phase unwrapping and temporal fitting.** *IEEE Trans Med Imaging* 2007, **26**:15–30.

6. Young A a, Li B, Kirton RS, Cowan BR: **Generalized spatiotemporal myocardial strain analysis for DENSE and SPAMM imaging.** *Magn Reson Med* 2012, **67**:1590–9.

7. Lai WM, Rubin D, Krempl E: **Chapter 3 – Kinematics of a Continuum**. In *Introd to Contin Mech*. *Volume 3*; 2010:69–153.
