# Supplementary material for: Comparison of left ventricular strains and torsion derived from feature tracking and DENSE CMR
Source: J Cardiovasc Magn Reson. 2018 Sep 13;20:63. doi: 10.1186/s12968-018-0485-4 (PMC6136226; doi:10.1186/s12968-018-0485-4)
Supplement: Supplementary file 4 — Validation using a Second Feature Tracking Software. (DOCX 302 kb) [file 12968_2018_485_MOESM4_ESM.docx]

*Validation using a Second Feature Tracking Software*

Data Analysis: Strain and twist were derived from the same subjects using SSFP images with a commercial feature tracking software (cvi^42^, Circle Cardiovascular Imaging Inc., Calgary, Alberta, Canada). Both endocardial and epicardial contours were manually drawn at end-diastole. The software automatically propagated the contours through the remaining frames, and manual adjustment to the contours was applied in the case of poor tracking to ensure visually acceptable contours. Slice-wise circumferential strain, longitudinal strain, and twist, averaged between endocardial and epicardial contours, were derived from output files generated by the software. Torsion and dyssynchrony were computed using the same calculations as DENSE imaging. Contour-based strains could not be derived from Circle because raw contours could not be exported from the software. In the supplemental tables below, ‘Contour Strain’ refers to the contour-based strains derived from ED and ES contours from TomTec. No adjustment to strain calculation was applied to Circle strains as we did for TomTec strains as the strain calculation method is not made available publicly for Circle.

Results: Circumferential strains derived from Circle showed good agreement with TomTec strains (Table S3-S4, CoVs=6–11%). Consistent with TomTec, Circle overestimated circumferential strains in all slices when comparing to DENSE (Figure S4), although the difference in mid-ventricular circumferential strain was not significant (-18.2 vs -17.2, p=0.07). Longitudinal strain from Circle was significantly overestimated compared to both TomTec and DENSE (by 4.6% and 4.9% absolute, respectively, both p<0.001). The agreement in longitudinal strains between Circle and DENSE was poor (CoV=22%, 95% limits: ±6.7).

Circle circumferential strains showed good agreement with contour strains, similar to the agreement with TomTec strains (Table S4). The bias in longitudinal strain (3.3%, CoV=18%) was smaller between Circle and contour strain than when comparing Circle to both TomTec and DENSE, but was larger than the bias between TomTec and contour strain. This is likely due to the potential difference in the calculation of strain by the Circle software compared to the way TomTec and DENSE calculate strain and also the inter-observer and inter-vendor variability in the contours.

Opposite to TomTec, Circle significantly overestimated torsion compared to DENSE (5.0 vs 3.5 deg/cm, p<0.001). Circle also significantly overestimated dyssynchrony (54.8 vs 16 ms, p<0.001). Both torsion and dyssynchrony had poor agreement with TomTec and DENSE as demonstrated by wide 95% limits and large CoVs (Figure S5). The poor agreement between Circle and TomTec demonstrated inter-vendor difference in feature tracking, and suggests that feature tracking cannot reliably track regional motion or motion parallel to the myocardial wall.

| **Table S3. Summary of comparison between Circle, TomTec and DENSE** | | | | | | |  |
| --- | --- | --- | --- | --- | --- | --- | --- |
|  | Circle | | TomTec^1^ | DENSE | P_1_ | P_2_ | |
| Circumferential Strain (%) | |  |  |  |  |  | |
| *Base* | -19.2 ± 2.8 | | -19.3 ± 3.3 | -15.2 ± 3.7 | 0.9 | <0.001* | |
| *Mid* | -18.2 ± 3.3 | | -17.5 ± 3.5 | -17.2 ± 3.4 | 0.22 | 0.07 | |
| *Apex* | -22.2 ± 3.3 | | -21.9 ± 5.7 | -19.4 ± 3.6 | 0.74 | <0.001* | |
| Longitudinal Strain (%) |  | |  |  |  |  | |
| *Four-Chamber* | -18.7 ± 2.6 | | -14.1 ± 4.3 | -13.8 ± 2.9 | <0.001* | <0.001* | |
| Torsion (deg/cm) | 5.0 ± 2.4 | | 2.1 ± 1.2 | 3.5 ± 0.9 | <0.001* | <0.001* | |
| Dyssynchrony (ms) | 54.8 ± 35.9 | | 42 ± 22 | 16 ± 20 | 0.06 | <0.001* | |
| ^1^Strains were adjusted for TomTec | | | | | | | |
| P_1_, Circle vs. TomTec; P_2_, Circle vs. DENSE  *Indicates statistical significance (P < 0.05) | | | | | | | |

| **Table S4. Bland-Altman analyses and coefficients of variation comparing Circle to TomTec, contour-based strains and the reference (DENSE)** | | | | | | | | | | |  |
| --- | --- | --- | --- | --- | --- | --- | --- | --- | --- | --- | --- |
|  | | Circle vs. TomTec | | | Circle vs. DENSE | | | Circle vs. Contour Strain | | |  |
|  | | *Bias* | *95% Limits* | *CoV* | *Bias* | *95% Limits* | *CoV* | *Bias* | *95% Limits* | *CoV* |  |
| Circumferential Strain (Absolute %) |  | | |  |  |  |  |  |  |  |  |
| *Base* | | 0.1 | ±4.2 | 6 | -4.0 | ±5.2 | 17 | -0.1 | ±4.3 | 6 |  |
| *Mid* | | -0.7 | ±4.7 | 7 | -1.1 | ±6.2 | 11 | -0.2 | ±6.6 | 11 |  |
| *Apex* | | -0.4 | ±9.0 | 11 | -2.8 | ±6.8 | 13 | -0.6 | ±9.1 | 12 |  |
| Longitudinal Strain (Absolute %) | |  |  |  |  |  |  |  |  |  |  |
| *Four-Chamber* | | -4.6 | ±8.7 | 24 | -4.9 | ±6.7 | 22 | -3.3 | ±8.8 | 18 |  |
|  | |  |  |  |  |  |  |  |  |  |  |
| Torsion (deg/cm) | | 2.9 | ±5.4 | 64 | 1.5 | ±5.0 | 36 | ----- | ----- | ----- |  |
| Dyssynchrony (ms) | | 14 | ±90 | 41 | 40 | ±56 | 80 | ----- | ----- | ----- |  |
| Adjusted strains for TomTec is used for comparison | | | | | | | | | | |  |
| CoV indicates coefficient of variation (%) | | | | | | | | | | |  |


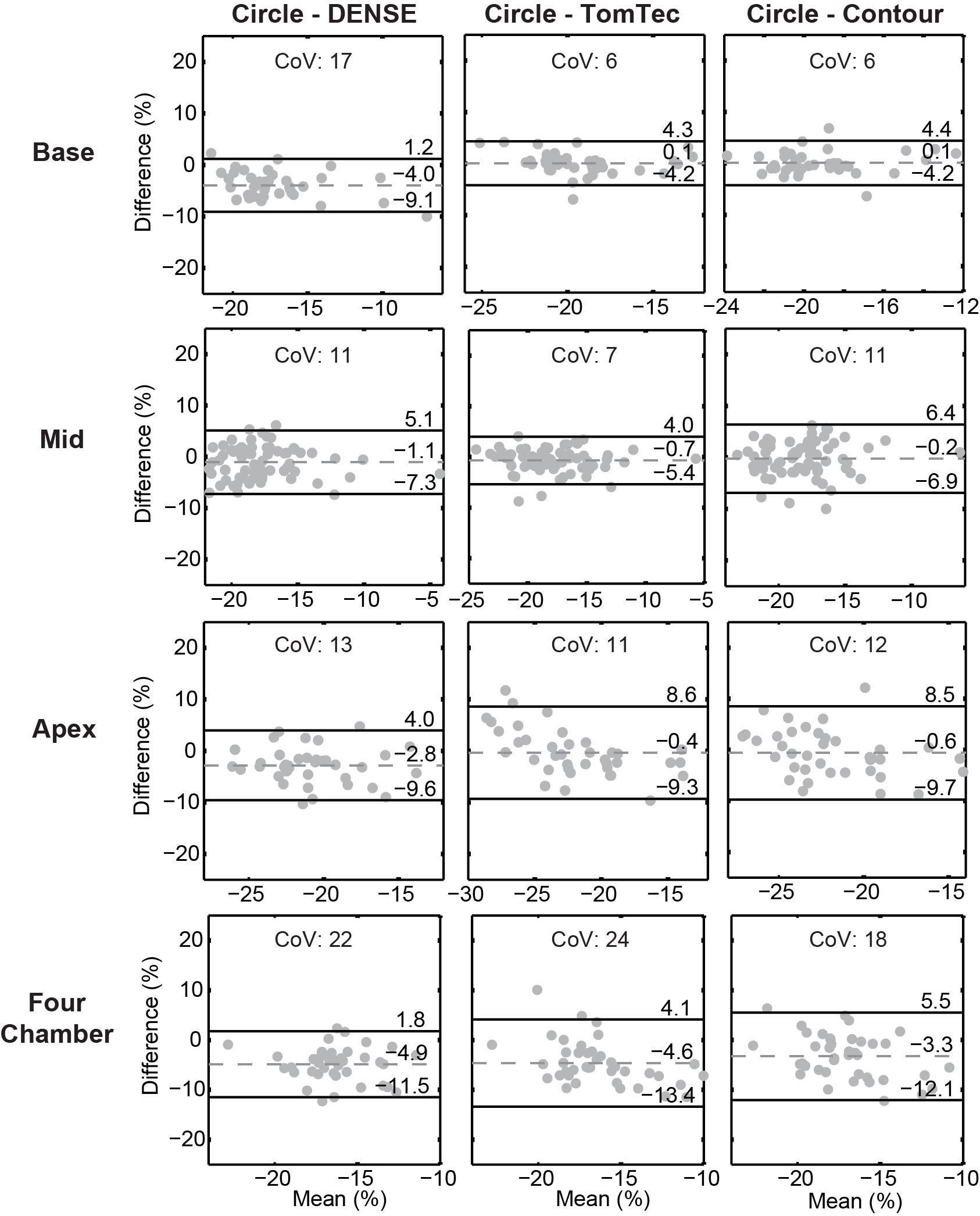


**Figure S4. Bland-Altman analyses for strains between Circle, TomTec, DENSE and contour-based strains.** All differences were calculated by subtracting the DENSE(left)/TomTec(middle)/Contour(right) strain from the Circle strain. The large negative biases indicate that Circle overestimates circumferential strain compared to DENSE for all image slices. Circle shows good agreement with TomTec and Contour circumferential strains while the longitudinal strain is overestimated by Circle.


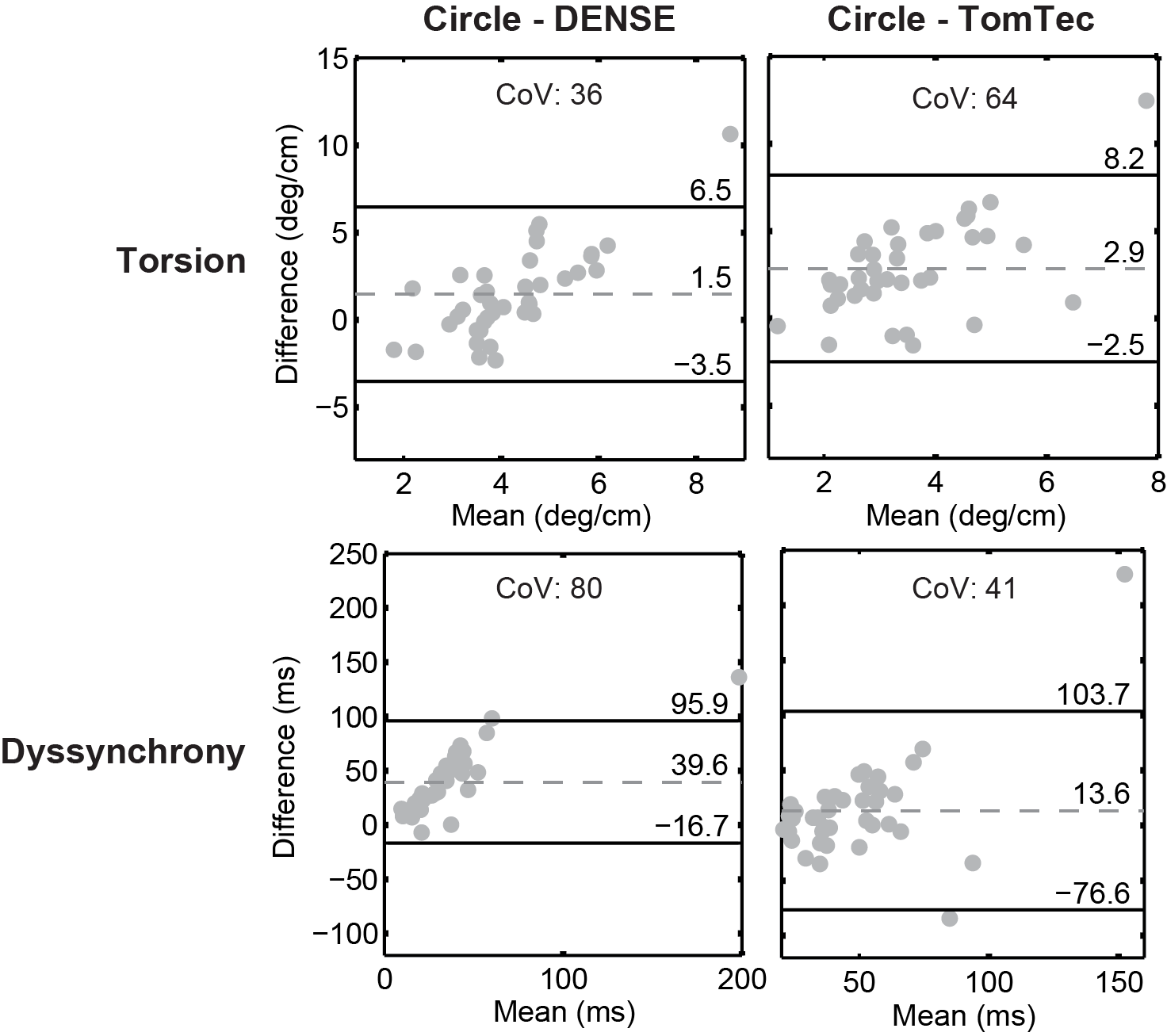


**Figure S5. Bland-Altman analyses for torsion and dyssynchrony between Circle, TomTec and DENSE.** All differences were calculated by subtracting the DENSE(left)/TomTec(right) measures from Circle measurements. The large biases and 95% limits indicate poor agreement between torsion and dyssynchrony from Circle and those from TomTec and DENSE.
